# Navigating Life With HIV as an Older Adult on the Kenyan Coast: Perceived Health Challenges Seen Through the Biopsychosocial Model

**DOI:** 10.3389/ijph.2023.1605916

**Published:** 2023-06-15

**Authors:** Patrick N. Mwangala, Ryan G. Wagner, Charles R. Newton, Amina Abubakar

**Affiliations:** ^1^ Centre for Geographic Medicine Research Coast, Kenya Medical Research Institute (KEMRI), Kilifi, Kenya; ^2^ School of Public Health, University of the Witwatersrand, Johannesburg, South Africa; ^3^ Institute for Human Development, Aga Khan University, Nairobi, Kenya; ^4^ MRC/Wits Rural Public Health and Health Transitions Research Unit (Agincourt), Faculty of Health Sciences, University of the Witwatersrand, Johannesburg, South Africa; ^5^ Department of Psychiatry, Warneford Hospital, University of Oxford, Oxford, United Kingdom; ^6^ Department of Public Health, Pwani University, Kilifi, Kenya

**Keywords:** older adults, sub-Saharan Africa, HIV, Kenya, biopsychosocial challenges

## Abstract

**Objectives:** This study explores the perceptions of adults living with HIV aged ≥50 years (recognized as older adults living with HIV—OALWH), primary caregivers and healthcare providers on the health challenges of ageing with HIV at Kilifi, a low literacy setting on the coast of Kenya.

**Methods:** We utilized the biopsychosocial model to explore views from 34 OALWH and 22 stakeholders on the physical, mental, and psychosocial health challenges of ageing with HIV in Kilifi in 2019. Data were drawn from semi-structured in-depth interviews, which were audio-recorded and transcribed. A framework approach was used to synthesize the data.

**Results:** Symptoms of common mental disorders, comorbidities, somatic symptoms, financial difficulties, stigma, and discrimination were viewed as common. There was also an overlap of perceived risk factors across the physical, mental, and psychosocial health domains, including family conflicts and poverty.

**Conclusion:** OALWH at the Kenyan coast are perceived to be at risk of multiple physical, mental, and psychosocial challenges. Future research should quantify the burden of these challenges and examine the resources available to these adults.

## Introduction

The last decade has witnessed a dramatic shift in the demographic profile of people living with human immunodeficiency virus (HIV) globally. Presently, many HIV clinics are caring for a growing number of adults aged ≥50 years (categorized as older adults) due to increased survival in people living with HIV (PLWH) and a steady rise in HIV diagnoses in this age cohort [1]. More than 30% of the PLWH in High-Income Countries (HICs) are now aged ≥50 years [1] compared to 15% in sub-Saharan Africa (SSA) [2]. These statistics herald a new era in the HIV epidemic response, where the needs and demands of the OALWH can no longer be ignored, especially in Eastern and Southern Africa, home to more than half the number of OALWH globally [1].

Research findings, mainly from HICs, indicate that OALWH present with an average of three comorbid conditions in addition to HIV, including medical diseases (e.g., diabetes, hypertension), mental health problems (e.g., depression, anxiety, substance use, cognitive problems) and social challenges (e.g., stigma, loneliness, and lack of social support) [3–6]. The observed mental health challenges reduce the quality of life of these adults and have important health implications, e.g., poor HIV treatment [7]. The physical health problems faced by OALWH may also be complicated by environmental and psychosocial challenges such as poverty, food insecurity and lack of support [7].

Despite the evidence of complex health challenges related to ageing and HIV, little research has qualitatively examined how OALWH understand their health and care needs. To date, most studies of ageing and HIV in SSA are cross-sectional studies focusing on biomedical processes and outcomes and rarely provide local insight into the health and wellbeing of these adults [8]. Qualitative studies are needed to better understand the experiences and needs of this diverse population, especially among low-literacy populations. This is especially important as many cohorts of OALWH are emerging for the first time across the SSA region, and the apparent variability in findings among previous studies, e.g., in the prevalence and determinants of chronic comorbidities [8]. Apart from complementing quantitative studies in accurately documenting the burden and determinants of the health challenges in these adults, qualitative studies will shed light on the contextual factors to guide the development or adaptation and subsequent implementation of culturally appropriate interventions in this population. Overall, the few qualitative studies among OALWH in SSA are mainly concentrated in Uganda [9–13] and South Africa [14–18]. Others are from Kenya, Eswatini and Malawi [19–22]. In Uganda, ageing with HIV is seen as a daily challenge financially and socially [9–13]. The key barriers to successful ageing with HIV in this setting include stigma, food insecurity, and unmet healthcare needs, particularly for associated comorbidities such as common mental disorders. In South Africa, the crucial barriers to living with HIV in old age include food insecurity, unemployment, stigma, and access to transportation and healthcare [14–18].

Emerging data suggest that OALWH in Kenya face complex challenges when seeking care, including visits to multiple providers to manage HIV and comorbidities, ageist discrimination, and inadequate social support [20, 21]. However, these data come from the Western region of Kenya. As such, the health and wellbeing experiences of OALWH from other parts of the country is not known. To bridge this research gap, we conducted in-depth interviews to explore the health challenges faced by OALWH at Kilifi, a low literacy setting at the coast of Kenya. Using the biopsychosocial framework, we explore the perceptions of 34 OALWH, 11 healthcare providers and 11 primary caregivers on the physical, mental, and psychosocial challenges of ageing with HIV in this setting.

### Theoretical Framework

**FIGURE 1:**
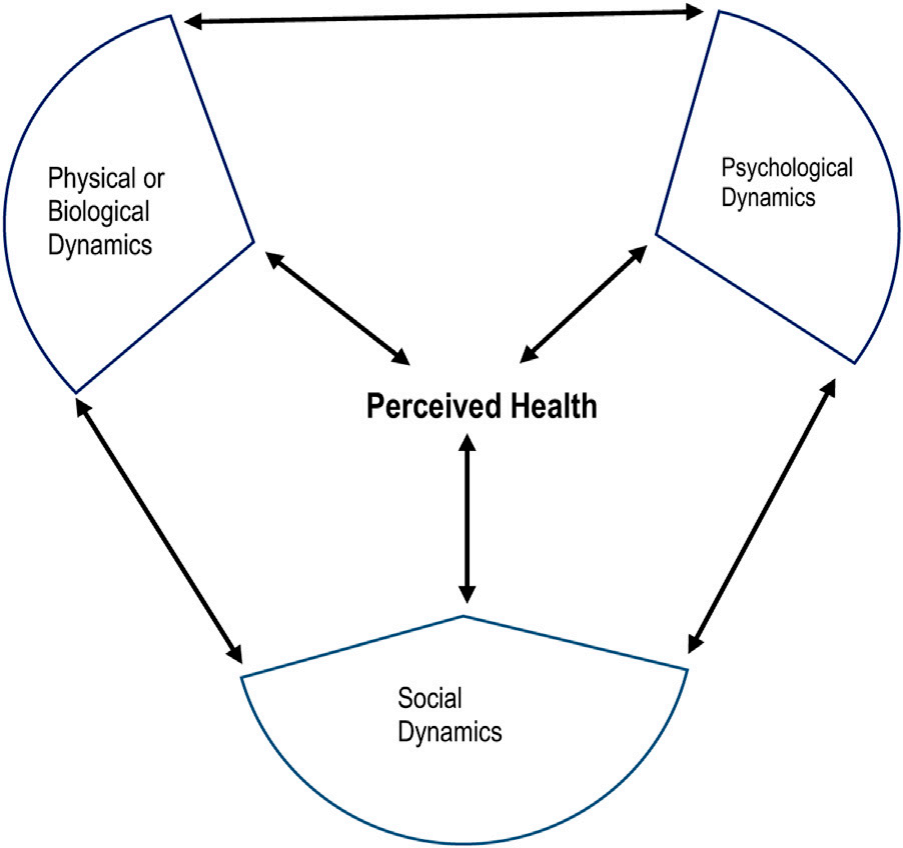
Components of the biopsychosocial model of health (HIV-Associated Neurobehavioral Disorders Study, Kilifi County, Kenya 2019).

We utilized Engel’s biopsychosocial model of health [23], which provides a logical account of the chronic, complex, and dynamic nature of HIV. This model recognizes healthy ageing as the ability to thrive in an evolving environment influenced by physical/biological, mental/psychological, and social factors (Figure 1). The model provides a holistic approach to understanding the health needs of older adults and is supported by calls for research that positively impact the physical, mental, and social aspects of ageing with HIV [24–26]. According to this model, the cause, manifestation and outcome of wellness and disease are determined by a dynamic interaction between physical, psychological, and social factors [23]. Each model component includes systems that reciprocally influence other dynamics in the model and also affect health.

## Methods

### Study Context and Participants

This study was conducted in 2019 at the Kenya Medical Research Institute (KEMRI) located within Kilifi County on the coast of Kenya. By the end of 2019, there were roughly 1.5 million residents in Kilifi County, the majority of whom were rural dwellers (∼60%), and 11% were aged ≥50 years [27]. Kilifi has an HIV prevalence of 38 per 1,000 people [28] in the general population and is currently unknown in those aged ≥50 years. People living with HIV usually receive care in specialized HIV clinics within primary care facilities.

This study involved the following three groups of participants.

a) OALWH aged ≥50 years receiving HIV care and treatment at the HIV Comprehensive Care Clinic of the Kilifi County Hospital (KCH).
b) Healthcare providers attending to OALWH.
c) Primary caregivers of OALWH.

### Recruitment and Eligibility

Study participants were selected purposively to represent diversity in the participants’ characteristics, including age, sex, and the cadre of service (for healthcare providers). Initial contact was made via a community health volunteer stationed at the KCH. Recruitment was conducted by a trained research assistant in liaison with the community health volunteer during their routine clinic visits. OALWH had to be ≥50 years old and on HIV treatment to be eligible. Primary caregivers were identified through the OALWH, usually during their scheduled clinic visits. These caregivers had to be directly involved in providing care and support, e.g., medical care to an OALWH. HIV providers were approached at their place of work and invited to participate. We specifically targeted providers who provided direct care to OALWH. Participants who agreed to participate were invited to a face-to-face interview at a convenient place, usually at KEMRI Kilifi.

### Data Collection and Tools

The lead author (PM) conducted in-depth interviews lasting, on average 45–60 min with each participant. All the interviews were guided by a pre-tested semi-structured interview schedule developed with guidance from prior work on HIV and ageing. All interviews were conducted in Swahili, English or Giryama. We also sought permission from the participants to take notes and audio record the interviews.

Among OALWH, topic guide themes included: patient illness experiences following diagnoses. These issues were explored using general open-ended questions, followed by additional probing to highlight further the issue raised. For healthcare providers, participants were invited to share their experiences providing care to OALWH. Among caregivers, we explored different issues, including their experiences taking care of OALWH and the challenges these adults faced in their environment. We also collected respondents’ demographic information such as age, sex, educational status, and employment status. For OALWH, we also collected HIV-related information. All participants were given Ksh 350 (about US$3) as compensation for time spent in the research, with transport expenses also reimbursed.

### Data Analysis

All the audio-recorded interviews were transcribed verbatim by a team of four trained research assistants. Subsequent data management was done using NVivo software (version 11). We applied the framework approach to analyze our qualitative data [29]. Initially, two authors (PM and AA) developed a preliminary coding framework inductively through in-depth reading of transcripts and deductively by considering themes from the interview schedules. These codes were discussed, and consensus reached on how they should be brought together into themes with guidance from the biopsychosocial model. The initial coding framework was progressively expanded to capture emergent themes as coding continued. When the coding was complete in NVivo, the lead author grouped all themes related to a specific concept to form categories and exported this to a word-text processor to produce charts. The generated charts were used to summarize data, look for similarities or differences and explore patterns among the three groups of participants in the analyzed data.

### Ethical Considerations

Written informed consent was obtained from all participants in the study. The study also obtained ethical clearance from the Kenya Medical Research Institute Scientific and Ethics Review Unit (KEMRI/SERU/CGMR-C/152/3804) and the Kilifi County Department of Health Services (HP/KCHS/VOL.X/171).

## Results

### Sample Characteristics

We interviewed a total of 56 participants (34 OALWH, 11 healthcare providers and 11 primary caregivers) in this study. Among OALWH, 53% were women; most (82%) had up to a primary level of education, and their age ranged from 50 to 72 years. All the OALWH were on HIV treatment. Healthcare providers included six registered nurses, two clinical officers, two project managers of community-based organizations and one HIV counsellor. All the primary caregivers were family members, most of whom (73%) were female. Further sociodemographic information and HIV-related characteristics of the OALWH are summarized in Table 1.

**TABLE 1:** Sociodemographic and clinical characteristics of older adults living with HIV (HIV-Associated Neurobehavioral Disorders Study, Kilifi County, Kenya 2019).

Characteristic	*n* (%) or median (IQR)
Median age	57 (54–63)
Female	18 (53%)
Educational level	
None	7 (20%)
Primary Level	21 (62%)
Secondary level	5 (15%)
Tertiary level	1 (3%)
Type of employment	
Unemployed/not working	12 (35%)
Small scale trader	15 (44%)
Casual worker	4 (12%)
Professional/skilled work	3 (9%)
Marital status	
Never married	1 (3%)
Married	19 (56%)
Separated/Divorced	7 (21%)
Widowed	7 (21%)
Living arrangements	
Alone	5 (15%)
Single generation household	4 (12%)
Multigenerational household	25 (73%)
Median household size	5 [2–9]
Residence (rural)	30 (88%)
Median duration since HIV diagnosis (years)	12 [10–15]
On ART treatment	34 (100%)
HIV status disclosure	
Non-disclosure	1 (3%)
Partial disclosure (to close family only)	18 (53%)
Full disclosure (beyond close family)	15 (44%)

Notes: ART, antiretroviral treatment; IQR, interquartile range.

### Perceived Biopsychosocial Challenges Confronted by OALWH

The different forms of biopsychosocial challenges faced by OALWH are summarized in Table 2. Overall, most of the OALWH shared their 10-plus years of experience living with HIV, from the time of diagnosis to their current state. The majority of the OALWH described moving from a state of shock, fear, anger, denial, hopelessness, or suicidal ideation upon diagnosis to a state of acceptance and ownership. This transition was not without challenges, as many of the OALWH noted going through traumatic experiences from family members, friends, workmates, and healthcare providers. Thereafter, they narrated moving from a state of ownership to a state of constant survival characterized by an array of health problems which we have conceptualized as physical (biological), mental (psychological) and social in the following sections according to the biopsychosocial model.

**TABLE 2:** Perceived forms of physical, mental, and psychosocial health challenges facing older adults living with HIV as discussed by study participants (HIV-Associated Neurobehavioral Disorders Study, Kilifi County, Kenya 2019).

Forms of health challenge	Number of in-depth interviews (among OALWH) where the health challenge was discussed (*N =* 34)	Number of in-depth interviews (among caregivers) where the health challenge was discussed (*N* = 11)	Number of in-depth interviews (among healthcare providers) where the health challenge was discussed (*N* = 11)
Physical complaints			
Comorbidities: chiefly ulcers/hyperacidity, hypertension, and diabetes	29	9	11
Wide-raging physical symptoms, e.g., pain/body aches, fatigue, insomnia	33	10	9
Functional impairments, e.g., difficulties walking, writing, holding things	18	3	3
Mental complaints			
Symptoms suggestive of common mental health problems, e.g., thinking too much, worry, stress, low mood, hopelessness/giving up easily	29	9	9
Cognitive complaints chiefly memory difficulties. Others including slow mental processing/learning, attention/concentration problems	22	7	4
Current drug and substance use, mainly “*mnazi*”’ and “*ugoro*,” which are the local palm wine and smokeless tobacco, respectively	5	0	9
Suicide and suicidal ideations	3	0	4
Psychotic-like symptoms, e.g., disorganized behaviour, aggression, lack of restraint and unwanted thoughts	1	3	3
Psychosocial complaints			
Financial difficulties, e.g., lacking school fees for dependents, walking long distances for HIV care	31	8	9
HIV-related stigma and discrimination especially internalized and enacted stigma	29	8	11
Ageism/ageist stereotype, e.g., neglect, verbal insults, vandalism	19	5	7
Isolation and lack of support	19	5	8
Loneliness	17	1	8
Food insecurity, e.g., skipping meals or going days without food	16	5	9

### Perceived Physical Health Challenges

Physical complaints were commonly described by several participants. Key among these concerns was the onset of multiple comorbidities in these adults, which was frequently associated with pain, loss of control of one’s body, emotional distress, and increased treatment burden. Hypertension, diabetes, ulcers/hyperacidity, hearing, and visual impairments were the most frequently reported comorbidities across the participants. Other conditions discussed (though to a lesser extent) included obesity, arthritis, stroke, teeth problems, cervical cancer, TB, and pneumonia. Apart from these comorbidities, somatic symptoms were also discussed in several interviews across the groups. Body pain, headaches, insomnia, fatigue or low energy, and limb numbness were also frequently reported. Some participants believed that the onset of the physical challenges was because of HIV-related factors (e.g., HIV infection itself, long-term ART), multimorbidity, old age, food insecurity, emotional distress, substance use, and the nature of someone’s work. Participant quotes are given in Table 3.

**TABLE 3:** Participants’ physical health challenges quotes (HIV-Associated Neurobehavioral Disorders Study, Kilifi County, Kenya 2019).

Perceived health problem	Examples
Multiple comorbidities	I am weak and constantly fatigued because I have ulcers, high blood pressure, and diabetes. My eyes are also painful and feel strained, and I sometimes experience toothache. As we are talking right now, my ears are not hearing well. So, all these diseases make me weak and significantly affect my wellbeing (*Female OALWH–10 years with HIV*)
	Ulcers is their main problem. Many of them complain of ulcers, and this is mainly because of stress. Anything small affects them. Sometimes you find one thinking too much and another telling you, “My children do not take care of me; they have forgotten about me” or “I have one child.” Someone tells you about their child who died long ago but is still affected. Stress is a big problem because they pile up issues, not because of HIV but other issues. Another issue is food. The elderly must look for food for themselves and their irresponsible children, who may be drunkards. Often, you find that they deal with their stress and that of their children (*Nursing officer*)
	Last year, my hands were painful, and I could not bend. I carried out an X-ray and was told it was arthritis. I was told there was no treatment, and the only relief was by massaging myself and using painkillers. So, I bought clove oil, and it is what I have been using. There are improvements, but the problem is not resolved yet. I am also unable to work, and to make it worse, and I have difficulties sleeping whenever I am stressed. I was recently diagnosed with high blood pressure but am not taking medications yet. I also have ulcers, but I do not usually keep it in mind (*Female OALWH–11 years with HIV*)
Somatic symptoms	My knees are painful. Sometimes even squatting is a big challenge. I also cannot rise too quickly from a seated position; I have to support myself. I must also use painkillers for some relief before I can work. This has affected me because I cannot work without them. Whenever the analgesic effect wears out, it becomes very difficult to use my legs. I also experience headaches sometimes, and my relief is usually the painkiller. These painkillers have become my life now (*Female OALWH–2 years with HIV*)
	My pace has become very slow nowadays. I feel heavy, and there is nothing I am lifting or carrying. I often feel tired, and, at such times, I prefer walking around for some time. I must do this to ward off intrusive thoughts. Since being diagnosed, I have also grown weak and lost my libido. In fact, whenever I start having sex, I have this sensation of my waist being cut with a razor (*Male OALWH–10 years with HIV*)
	My husband cannot do heavy manual jobs. He used to work at the sea at night, which is usually cold and requires energy. It reached a point I had to stop him from going there anymore because he was weak and felt he was risking his life. Before this, he often used to complain of being sick. I was not at peace. I found it pointless for him to earn money that way and end up using it to medicate him. So, I told him to stay at home and do anything else. We are living by the mercies of God (caregiver)

### Perceived Mental Health Challenges

We have discussed these issues under the following headings: symptoms suggestive of common mental health problems, cognitive symptoms, substance use problems, and others (suicidal ideation and psychotic symptoms). Participant quotes are given in Table 4.

**TABLE 4:** Participants’ mental health quotes (HIV-Associated Neurobehavioral Disorders Study, Kilifi County, Kenya 2019).

Perceived health problem	Examples
Symptoms of stress, depression, anxiety, trauma	Living with HIV is difficult, my brother because you must take medications daily without missing and must live knowing that you are sick and could die any moment. This reality makes me depressed. I also do not know who will take care of my family if I die because my relatives are useless to me! I am jobless, and this makes me very sad and depressed because I have a family that needs to be cared for! I do not have money, getting food is a big problem, and to tell you the truth, I cannot even afford to pay school fees for my children. I must borrow to survive. As we are speaking, I have a court case because am unable to repay a loan I took (*Male OALWH–20 years with HIV*)
	My eyes cannot see well and are very itchy. Today in the morning, I had to compress them with hot water. The itchiness is often accompanied by coughing. I was prescribed some medications, but they were not helpful. You see this paper am holding; I cannot read anything on it unless I view it in very bright light during the day. At night, I can hardly make out the person calling my phone! This has affected me a lot. I am worried and do not know when this problem will end. Sometimes I want to work, e.g., write something, but I cannot. At night I must also depend on someone else to guide me. Other times I cannot work at all because of my eyesight problems. Whenever I am depressed, I just run to God (*Female OALWH–2 years with HIV*)
	My mum is generally stressed and very irritable. Sometimes she can get so stressed that she falls sick, forcing us to take her to the hospital (*Male Caregiver*)
	Sometimes, she gets very preoccupied with thoughts until she cries … not often, but it happens. Sometimes she also has trouble sleeping, and when I inquire why she tells me, “I am always thinking, and I do not know exactly what I am thinking about, and I do not have peace.” I think she would love to be free with everyone and go anywhere she wants without people talking behind her back. She is sensitive, you know. When she hears people laughing, she usually asks me, “Why are they laughing? Are they laughing at me?” Sometimes, she does not come out of her room for two or 3 days and must force her to eat something (*Female Caregiver*)
	There is this client; her skin has spots, her jaw has dropped, and looks weak when she walks. She is often in deep thought; for instance, we could be in a seminar where many people participate, but you find her lost in her thoughts. We had to talk to her at some point, and we realized she had been abandoned by her husband, who had married another wife. We had to do a referral for this client because of these issues (*Project manager of a community-based organization*)
	You know these people are dealing with so many things which affect them. Stress, anxiety, and depression, and that is why screening is being done, and any positive case is linked with the psychiatric clinic (*Nursing officer*)
Cognitive symptoms	I can plan to do one or two things but later abandon them and do something different without any reason. For instance, I could be having a meeting, perhaps with some elders, but end up not doing and instead do other things. There is also this issue of forgetting, which is like a disease to me. I usually have a problem finding my things. Like yesterday, I could not find my money. I am worried about this problem, which is like a disease nowadays. I start doing one thing, then abandon it, and do the same to another. Sometimes my food gets burnt in the kitchen because I forget I was cooking and start doing other things. One time I decided to see a clinician about this disease because it was too much. I do not know whether it is because of these medications we are taking or something else. The clinician told me it would end with time. One thing I know, though, is that I was not like this before. This problem started when I began taking these medications. I am very surprised! I am not alone in this; many of my peers also complain of the same thing. Or could it be old age (*Female OALWH–13 years with HIV*)
	I can place my things in one place and forget about them. This can be money or even fruits. If I do not tell my husband that I have bought, e.g., fruits and have placed them in the cupboard, those bananas will go bad. He (my husband) will remember, but I will not. My body also becomes numb sometimes. My hand becomes heavy and immobile, especially in the morning. This extends to the shoulders and usually makes it impossible to carry things. The hands sometimes tremble and experience prickly feeling (of pins) in addition to numbness and general weakness. One of the legs is also involved in this (*Female OALWH–10 years with HIV*)
	To speak the truth, my husband does not concentrate nowadays. Actually, the real issue is his forgetfulness. It is a real concern for me. I have tried to make him understand that he has this challenge, but he never accepts it. I have suggested we look for medications, but he does not want to. He has also become very irritable lately. I think very few people will be able to tolerate him (*Female Caregiver*)
	You might explain something to one of them (OALWH) and moments later find that they contradict that same information. We have such cases, but not very many. Some take their ARVs and septrin/co-trimoxazole the same way when it is not supposed to be. In fact, some interchange these drugs when they come for their refill appointments. Because of this, we usually emphasize how the drugs should be taken and give them shorter appointments, e.g., weekly, for those who stay nearby. We also ask them to come with their treatment support persons, but some say they do not have them (*Nursing officer*)
Substance use and suicide	My only addiction is to smokeless tobacco, but I stopped taking alcohol long ago. I have never smoked cigarettes before. I must use it (smokeless tobacco) every morning; otherwise, my heart will not be at peace; I will start feeling sad and not concentrate on any task. I only feel okay once I use it (*Female OALWH–2 months with HIV*)
	Adults of my age in this community are ill-mannered. They like taking alcohol and smoking cigarettes. They also have extra-marital affairs. Every morning, they must have a packet of cigarettes and ensure they do not miss a bottle of the local palm wine because they cannot afford beer. They have to take a bottle or two for them to sleep well (*Male OALWH—14 years with HIV*)
	I often feel like taking my own life. These thoughts come to me quite often, especially when I am stressed. Usually, a certain voice tells me that I am better off dead than alive, and another voice tells me not to do it. There is a fight between God and the devil. Sometimes I wake up in the middle of the night around midnight and start thinking about many things, which is how I lose my sleep entirely. I am just thinking, “I do not have money, my wife is unemployed, and my children are still in school. Where will I get food?” So, in the morning, I share this with my wife. At the church, we are also told that suicide is not the solution; it is adding the problems, so it is better to struggle but survive (*Male OALWH–14 years with HIV*)
	Not just suicidal ideations. Sometimes we also have cases of actual suicides. Last month, an older adult committed suicide. He was recently diagnosed with HIV, and two of his three wives also turned positive. We do not know what happened, but we received news that the old man had committed suicide 2 days later. So, it is a challenge (*Clinical officer*)

#### Symptoms Suggestive of Common Mental Health Problems

Emotional and behavioral symptoms, suggestive of anxiety and depression, were described to be prevalent across the participant groups. Key among these symptoms included thinking too much, persistent stress, low mood, anger/irritability, worrying a lot, hopelessness, restlessness, panic attacks, and nightmares. Other prominent symptoms included insomnia, self-isolation, low self-esteem, headaches, and low libido. Overall, these symptoms were described to be on-and-off among the OALWH and were frequently associated with the exacerbation of physical conditions, chronic use of drugs, financial instability, and overall poor quality of life. For many participants, the onset and persistence of physical body changes (e.g., peripheral neuropathy, obesity, body weakness) were considered a significant risk for mental complaints.

#### Cognitive/Neurological Challenges

The commonly reported complaint in this category was memory difficulty, e.g., difficulties recalling important dates or finding items. Surprisingly, most OALWH reported that they rarely forgot to take their medications. Many healthcare providers corroborated this, although they also noted that there are some OALWH at risk of poor adherence caused by cognitive impairments. Other cognitive symptoms included attention/concentration, processing/learning, movement, and multi-tasking difficulties. Peripheral neuropathy was also reported to be a common neurological problem in these adults and was associated with significant distress. Among OALWH, the cognitive symptoms were viewed as an additional disease, and many did not understand its causes. The few who sought medical attention ended up being disappointed, sometimes being advised that it will resolve on its own or there is medicine, but it is costly.

#### Drugs and Substance use Problems

Current drug and substance use was mainly discussed by healthcare providers. In contrast, most OALWH reported having a history of drug and substance use, especially alcohol and tobacco but stopped following HIV diagnosis. However, some confessed to being social drinkers. “*Mnazi*” and “*ugoro*” (smokeless tobacco or snuff) were the commonly used substances. Many providers emphasized that *mnazi* drinking is a big problem, noting that some OALWH came for their routine clinic appointments while drunk and sometimes forgot to take their ART drugs. Some providers also associated the *mnazi* drinking problem with unsuppressed viral loads, treatment default, and sexual risk-taking behaviors, e.g., multiple partners. The key underlying factors for the increased use of *mnazi* included family conflicts which often led to stress, unstable sexual partners, negative coping skills, culture, and easy access to these substances. *Ugoro*, the other commonly abused substance, was especially common among women. Beer, cigarettes, khat and marijuana were also discussed, although to a lesser extent.

#### Suicidal Ideation and Psychotic-like Symptoms

Three OALWH reported having intermittent suicidal ideations. This was corroborated by the healthcare providers. The commonly associated factors included persistent stress (caused mainly by financial difficulties and family conflicts), evil spirits, and HIV-related (e.g., getting tired of taking ART and not getting healed). Though limited, there were also few reports of psychotic-like symptoms (e.g., disorganized behaviour, aggression, lack of restraint and unwanted thoughts) across the three groups of participants. These cases were frequently observed around the time of HIV diagnosis.

### Perceived Psychosocial Challenges

We present these issues under the following categories: a) financial challenges, b) stigma and discrimination, and c) loneliness and isolation. Participant quotes are given in Table 5.

**TABLE 5:** Participants’ psychosocial challenges quotes (HIV-Associated Neurobehavioral Disorders Study, Kilifi County, Kenya 2019).

Perceived health problem	Examples
Financial challenges	I am jobless, my brother. I have also lost my hands-on skills and experience with time. I am weak and do not have money. Getting food is a problem, and to speak the truth, even raising school fees for my children is a huge problem. Nevertheless, I have a very good friend, a lawyer, who is of great help to me. Sometimes, I eat at his place, lunch and dinner, but he does not know I live with HIV. Sometimes I am depressed and anxious because I do not know how to provide for my family. My relatives are of no help to me. Like yesterday, we did not have dinner, and I had to borrow the fare to come to this place. As we speak, I am in debt and face a court case because I cannot repay a loan. Often, I sleep hungry and must borrow to survive (*Male OALWH–20 years with HIV*)
	To speak the truth, I do not receive any support from my relatives, and this is not because there are no people to help. My husband and father died. I often sleep hungry. Sometimes, I only have a cup of porridge for three consecutive days … and this is usually borrowed from a neighbour so that I can take my medications. Other times, I survive on a cup of tea or hot water. Fare is also a big challenge. However, whenever I lack fare, I usually walk to the clinic from Bamba to Kilifi (around 50 km). I usually start my journey 2 days ahead of my clinic appointment and have two rest points in between, first at *Magogoni and* then at Kilifi at my uncle’s place. From my uncle’s place, I then walk to the clinic very early in the morning before 6 am (*Female OALWH–5 years with HIV*)
	Financial challenge is a big problem for these adults because they are neglected by their children sometimes. The day I am on duty at the clinic, I usually give many of them fare. You will hear many saying, “sister, please help me with fare, I walked to the clinic, and I have to walk back.” If you have a good look at them, they appear exhausted, so you end up helping them. Even food is a challenge. In our discussions, the majority say that they have one meal per day and mind you, some fail to take their medications whenever they miss a meal due to dizziness claims (*Nursing officer*)
	The other day, we visited an elderly client in the community, a woman caring for two grandchildren living with HIV. The grandmother is unemployed, and the children’s mother is not around. The grandchildren are in school. They need support with their schooling in addition to food. She is largely unable to provide all these and often must borrow. Sometimes, you find that even the skills you give them, e.g., rearing chicken or fish and making baskets, are not helpful because they are old and unable to work. In such cases, we assist them with food, counselling sessions, linkages, and referrals to foster treatment adherence and the wellbeing of the children (*Project manager of a community-based organization*)
	I usually borrow money from neighbours or friends to buy food whenever my mother does not have. I do not mind the debt because my target is to ensure that my mother has food to take her medications. However, it is a bit stressful because sometimes she sleeps hungry. And this is complicated by my father, who often quarrels my mother over small issues, increasing her stress. Every time my mum asks for money, he says he does not have it, and if we ask him how we will survive, he replies, “Can’t your son look for the money?” It is frustrating because how can I take up this responsibility while in school? In fact, the one educating me is usually my grandfather (*Caregiver*)
Stigma and discrimination	The elderly living with HIV undergo many challenges in the community. For instance, you find one is sick, and no one is taking care of him at home. In fact, people will avoid interacting with this person, especially where he sleeps, out of fear of contracting the virus. Some of them have called me seeking help. Sadly, when I go to their homes, I find some have not even bathed for a whole week! In such a case, I usually clean up the person and assist him in eating. It is sad because the family places the food there; the person is sick and can hardly eat alone! Some are told to stay with their HIV because they know where they took it from! As we are speaking, there is a woman I would like to talk to because she stays in Malindi but takes her ART medications from Nakuru because she does not want relatives to know that she is living with HIV. So, HIV education has come for sure, but it is far from being complete (*Female Peer counsellor*)
	She is often in a low mood. She gets offended whenever she hears people talking and laughing on the other side. I think she would really like to be free and move about without people badmouthing her. (*Caregiver*)
	For men, there is a challenge, a huge challenge. Getting them is a very big challenge. We have tried to work with male champions to convince their fellow men to join us. Stigma is a big issue. In our meetings, you mainly find the ladies coming, and when you ask them about their husband’s whereabouts, they often tell you, “He has gone to work and has sent me to listen to the messages on his behalf.” So, they have that fear; they do not want to be seen. The case is not different in the primary facilities because many of them are not coming. He either sends the wife or comes but is very careful not to be seen, e.g., comes in through the back. Because of this fear, you find that some of the men do not want to be tested. We have several cases where the wife is living with HIV; the husband got tested and turned positive but is still in denial. You will find the wife saying, “We went and got tested with him but has insisted that the machines were not functioning and as such, he is not infected.” Others also note that their husbands have constantly refused to start ART and use condoms, and whenever these issues are brought up, they end up quarrelling (*Project manager of a community-based organization*)
	That is a big problem (stigma). Ours is a small facility, so we schedule 1 day in a week to see the clients living with HIV. Surprisingly, even the community knows Tuesday is the day for those taking ARV drugs. So, most of them come very early in the morning. Therefore, I must come in very early to serve those who have stigma. Alternatively, that person will hang around the clinic from morning till evening when everyone has left so that people will not understand what she is doing there, or you will find that they will skip their appointed clinic dates (*Nursing officer*)
	Some of them are mistreated when they come for their appointments until I get angry! Let’s say you are late for your appointment; you will be shouted at, insulted, and reprimanded like a child. In fact, some end up crying and I have to calm them down. It is sad because the culprits are usually the nurses who often shout at them, saying, “Take your drugs, or you will die of AIDS alone, and you will not see us!” It is shocking because some of them are treated very badly to the point that I am also angered. Some come to my desk, shedding tears, so I have to calm them down. At some point, it was too much that some of them transferred to other clinics (*Adherence counsellor*)
	The elderly are being burned alive and cut with pangas. As a matter of fact, some of my neighbours recently cut their elderly aunt severally with a panga because of witchcraft accusations. Later, their father also passed through the same ordeal by one of his children … the head was completely slashed off with a very sharp panga! And the reason is always witchcraft; I have not heard of any other thing. Such cases are numerous where I am living. I have witnessed four elders being cut with pangas, and the sad part is that these people will never expose each other to the police … even if I know it is you who did it, I will never say it. Even when the police come, the locals will never expose the culprit (*Female OALWH–12 years with HIV*)
Loneliness and isolation	I desire to have an exclusive male companion my age to spend the rest of my life with. Having one who is already married is problematic because sometimes I am very lonely and would want to call him, but I end up avoiding it out of fear, “What if he is with the wife? And you know it is not good to break someone’s marriage.” Because of such challenges, I would like to have my own man who does not belong to anyone, one I can relate to freely and love fully. You know, with someone else’s husband, the love is incomplete, and it feels like I must hide constantly, and it reaches a point I feel afraid. Sometimes he does not answer my calls and sends a text message saying he is at home. So, you see, with such problems, I just want to get my own man who will live with me, someone I can talk with, especially when things are difficult because it is stressful at times (*Female OALWH–11 years with HIV*)
	Sometimes, I wish I could have friends with whom I can confide and share my challenges, but I am afraid of being discriminated. You cannot trust anyone. I have many friends, but they are all fake (*Male OALWH–20 years with HIV*)
	There are times he isolates herself from other people. You might see her seated alone and in deep thought. Sometimes she wakes up and begins to shed tears. She usually feels that her life has come to an end, perhaps (*Caregiver*)
	Whom will I share my problems with? I do not have anyone. Anyone I open myself to will want to know how and why my husband is facing those challenges. What is the cause? This will mean I start discussing and revealing the issues of my husband, which is not good (caregiver)

#### Financial Challenges

This was discussed in virtually all the interviews we conducted. Many participants felt that financial difficulty was the most crucial issue affecting OALWH in the study setting because of its massive impact on food security, caregiving responsibilities, emotional wellbeing, and management of HIV and other comorbidities. Many OALWH reported skipping meals or going hungry for some days, lacking school fees for their dependents, and walking long distances to access HIV care for lack of money. This was corroborated by the healthcare providers and caregivers, who further stated that many of those who slept hungry tended to skip their medications, complaining of dizziness, headaches, and stomach discomfort. To some extent, this was associated with poor treatment outcomes, including unsuppressed viral load and poor retention in care.

#### Stigma and Discrimination

This theme incorporates HIV-related stigma and ageism (discrimination based on age). Despite relatively high levels of disclosure, many OALWH experienced HIV-related stigma. Discriminatory behaviour (enacted stigma from malicious gossip to outright discrimination, e.g., neglect, isolation, verbal insults) was reported to be common, especially in the most rural areas of the study setting. The perpetrators were mainly family members. Stigma was also reported to be common in mnazi drinking dens called “mangwe,” burial ceremonies, and HIV clinics. Internalized stigma emerged as an important theme, especially among the OALWH and was frequently associated with self-isolation, anxiety or high consciousness of self, fear of seeking assistance, attending very far HIV clinics, stress, and irritability.

Many participants also highlighted discrimination based on age, which was perceived in multiple settings, including the home (mainly through isolation, neglect, and lack of respect from children), HIV clinic (e.g., scolded openly and verbal insults), and the community level. It also emerged that some community members around the study setting (especially in the most rural areas) regarded older people suspiciously. Many participants narrated hearing or witnessing several older people being beaten, beheaded, or burned alive in their houses for suspected witchcraft, and the perpetrators were mainly close family members.

#### Loneliness and Isolation

This emerged as an important theme in the conversations with OALWH and healthcare providers. Despite living in multigenerational households, many of the OALWH expressed loneliness and isolation. Some had lost their partners, some their closest relatives and others saw their circle of friends getting smaller. Still, others felt neglected by those around them.

## Discussion

### Summary of Key Findings

We conducted this study to gain a preliminary understanding of the health challenges facing OALWH on the coast of Kenya. Our study provides insight into the complex challenges of ageing with HIV and opens up opportunities for further epidemiologic research and subsequent development of tailored interventions for OALWH. Overall, our findings reveal that OALWH in this setting are particularly vulnerable to mental health problems, especially symptoms suggestive of common mental conditions. They are also at risk of physical health challenges, including comorbidities and somatic complaints. Mental and physical health impairments are complicated by psychosocial challenges, including poverty, lack of support, stigma, and discrimination. Noteworthily, there was an overlap of perceived risk factors across the three health domains (e.g., family conflicts, poverty, food insecurity), suggesting that OALWH who experience these shared cumulative risk factors are more likely to face multiple health challenges. It also implies that the action taken to mitigate any or some of the shared enabling factors is likely to have a preventive spillover effect across multiple health domains. Most of the views of OALWH on health challenges were corroborated by views from the providers and caregivers. However, a few disparities emerged in some of the perceived health challenges. Current drug and substance use, for instance, was reported mainly by healthcare providers, while cognitive complaints were discussed by OALWH.

### Physical Health Challenges

Our discussions clearly showed that physical challenges are important concerns for older adults living with HIV on the coast of Kenya, given their negative impacts on other health domains and overall health. As the number of OALWH increases in many HIV clinics, HIV care will increasingly need to draw on a wide range of medical disciplines besides evidence-based screening and monitoring protocols [30]. Unfortunately, most healthcare providers presently lack guidance and training to identify and manage declines in physical and mental capacities in this population. The siloed provision of HIV care and other comorbidities could also imply that providers are unaware of the patients’ other conditions. Our findings are similar to those reported in a recent qualitative exploration of challenges seeking HIV care services for OALWH [20]. Integration of services for HIV and non-communicable diseases in primary care may enable settings like Kenya to expand healthcare coverage for PLWH.

The current findings also revealed a prominent intersection between ageing and HIV. For most participants, ageing rather than HIV was the primary concern. This is not surprising considering that among PLWH with controlled viraemia, HIV infection often stops being the overriding comorbidity but is simply a key element in the overall milieu of multiple conditions [31, 32]. In a few instances, however, participants discussed that their experiences were associated with HIV, long-term medication use, or side effects, although their providers attributed these conditions to normal ageing. They advised the OALWH to “wait,” and they will improve with time. Disagreements about the cause of a symptom or health condition may contribute to doubts about the effectiveness of treatment or conceal an emerging disease and contribute to delayed diagnoses such as medication side effects and polypharmacy.

### Mental Health Challenges

Our finding of substantial symptoms suggestive of common mental health conditions among OALWH is consistent with previous reports of poor mental health among PLWH in the study setting, albeit among younger populations [33, 34]. However, our study does not establish whether the burden among OALWH is higher or lower than that observed among young PLWH. Quantitative studies are needed to confirm this comparison. Nonetheless, OALWH may be facing a higher burden of common mental problems than their younger counterparts for different reasons. Firstly, many of the longest surviving OALWH may be significantly impacted by the legacy of the early years of the epidemic, including multiple bereavements and “survivor guilt” [35]. Secondly, the higher burden may also be attributed to the numerous challenges that OALWH face, e.g., poverty, food insecurity, caregiving responsibility, double stigma, and the onset of physical body changes, as evidenced in our study.

Since the beginning of the HIV epidemic, the manifestations of cognitive and neurological problems have been ubiquitous and frequently associated with poor treatment outcomes and impairment of activities of daily living [7]. In our study, the most frequently reported cognitive problem—memory difficulty—was commonly associated with stress and shame. Strikingly, healthcare providers seldom suspected cognitive impairments among OALWH and screenings were never done. This observation is similar to what has been reported in South Africa [32, 36]. Despite the reported memory challenges in this study, OALWH rarely forgot to take their medications (from their own self-reports and that of their providers). This finding is not unique in the HIV literature [37]. It is possible that OALWH are more organized and experienced and possibly more motivated after experiencing the initial devastating outcomes of the HIV pandemic. However, it is still essential to monitor the cognitive function of these adults to prevent treatment non-adherence, considering the multiple challenges they face, which are likely to impact their cognitive function and worsen as they grow older.

Our findings also noted a section of OALWH at risk of substance use dependence, especially home-brewed alcohol, called mnazi. This is not surprising given that the consumption of mnazi is common among the inhabitants of Kilifi because it is cheap and often less regulated [38]. The need to address this problem is even more crucial since it was associated with rising cases of sexual risk-taking behaviour and poor treatment outcomes in this cohort.

### Psychosocial Challenges

Psychosocial factors are well-known predictors of treatment adherence, disease progression and quality of life for PLWH [39]. Findings from our exploratory study suggest that financial difficulties, loneliness, stigma, and discrimination are prevalent among OALWH in the study setting and are associated with mental complaints and physical health problems. Similar findings have been reported in Western Kenya among OALWH [20]. These findings are not surprising, given the country’s prevailing situation of older adults. According to Help Age Kenya, the majority of older people in Kenya, especially those in rural areas, live in absolute poverty [40]. A recent report, the National Gender and Equality Commission Report, dubbed “Whipping Wisdom,” also established that older adults in Kenya faced various forms of violence, including social stigma, neglect, abandonment, and hindrance from using and disposal of property [41].

### Implications

Our study highlights opportunities for interventions and further research. It is important to reiterate that the health challenges faced by OALWH at the coast of Kenya are often interconnected and require a cohesive and collaborative response to achieve maximum benefits. Such interventions should target modifiable factors such as emotional support and integrate needed social and community support, e.g., case management services, food and nutrition support, financial assistance with caregiving responsibilities, and transportation. Context-specific interventions to help OALWH develop and nurture their own coping strategies are also critical in this population. Patient-centred care and patient self-management principles (e.g., self-reliance and empowerment) are critical elements in chronic care and are advocated as universal strategies in international frameworks of chronic care [42]. Integrated care models—which focus on the holistic view of the person by considering both medical and psychosocial needs, e.g., comprehensive geriatric assessment, are likely to improve the patient situation and the treatment outcomes. While OALWH are the primary target of most of the existing interventions in this cohort [43–45], research is required on how to build the capacity of healthcare providers, family members who act as informal caregivers and friends to provide support and care to those ageing with HIV. Future research should also examine the resources and resilience among these individuals to fully understand the vital role of resilience in empowering OALWH to enact processes that buffer health from the identified stressors. Future research is also needed to quantify the existing burden of physical and mental challenges in this population in Kilifi and confirm the risk and protective factors of these challenges. Furthermore, there is an urgent need for research to pilot and test the applicability and effectiveness of interventions underlying the determinants of physical and mental impairments in this setting.

### Strengths and Limitations

To our knowledge, this is the first study to richly explore the health challenges of OALWH on the coast of Kenya and among the few studies in Kenya. Unlike previous studies, a key strength of this work is that participants comprised a diverse group of stakeholders. This ensured that the views were diverse and contrasted across participant groups. Using a biopsychosocial model helped us obtain a richer insight into the complexity of the identified health challenges. However, our findings emanate from a predominantly rural setting, and circumstances may differ from those in urban areas. Only OALWH who were on long-term HIV treatment were interviewed in this study; thus, their circumstances may also differ from those who are not in care or the newly diagnosed. As is the norm for qualitative studies in general, data collection, analysis, and interpretation are subject to individual influences and researcher biases; nonetheless, we countered this effect by maintaining reflexivity and constant discussion with the research team to provide rigour and credibility to the study.

### Conclusion

Our findings provide initial insight into the biopsychosocial challenges confronted by OALWH in a low-literacy Kenyan setting. The participants’ views indicate that mental complaints (especially symptoms suggestive of common mental health conditions and memory difficulties), physical problems (particularly comorbidities and somatic symptoms) and psychosocial challenges (especially poverty, stigma, and discrimination) are of concern among OALWH. Many of the perceived risk factors for these challenges often overlap across the biopsychosocial domains. Our study also highlights several opportunities for interventions and future research to tackle these issues in the study setting. Future research should quantify the burden of these challenges, examine the resources available to these adults, pilot, and test feasible interventions in this setting, and in doing so, aim to improve the lives of older adults living with HIV.

## Author Disclaimer

The views expressed in this publication are those of the author(s) and not necessarily those of AAS, NEPAD Agency, Wellcome Trust, or the UK government. For the purpose of Open Access, the author has applied a CC-BY public copyright license to any accepted manuscript version arising from this submission.
